# Antibiotics: Responding to a Global Challenge

**DOI:** 10.3390/antibiotics1010014

**Published:** 2012-06-14

**Authors:** Christopher C. Butler

**Affiliations:** School of Medicine, Institute of Primary Care and Public Health, Cardiff University, 5th Floor, Neuadd Meirionnydd, Heath Park, Cardiff CF14 4XN, UK; Email: ButlerCC@cardiff.ac.uk; Tel.: +1-514-913-1983; Fax: +1-514-504-2077

## Editorial—Antibiotics: A New Journal Rising to a Global Challenge

### Antibiotics—A Short-Lived Miracle?

The miracle of antibiotics is hard to exaggerate. Each day, in every corner of the world, antibiotics improve, or could be improving outcomes in the septic neonate, the child with pneumonia, the new mother after a complicated delivery, the patient undergoing surgery, the nursing home resident with a urinary tract infection, the patient being treated of cancer, or the trauma patient on life support. The miracle also keeps our animals healthy for effective food production.

But the miracle of these ‘wonder drugs’ is under threat and may be short lived: antimicrobial resistance is relentlessly increasing, especially for Gram negative organisms, prompting the oft expressed concern that we are plummeting head-long back into the pre-antibiotics era where clinicians and families once again will have to stand by and watch patients and loved ones die from once easily-treated infections. 

### Antibiotics: The Journal’s International, Multi-Disciplinary and Broad Focus

One thing is clear: there is no single, simple, magic bullet that will solve this problem. Clearly, multidisciplinary, creative responses are required, making antibiotics one of the most exciting, challenging, and fulfilling fields to be researching. 

To name but a few areas, we need more urgent and clearer focus on:

- Biomedical innovation, capitalizing on the tantalizing promises of genomics and personalized medicine for new agents that are better targeted to individual host and pathogen characteristics to achieve improved clinical outcomes.
- Better diagnostics, especially those that are useful at the point of care to guide clinical decision making about *whether* and *what agent* to prescribe. These diagnostic tests should be affordable and feasible also in resource-poor settings.
- Enhanced understanding of how antibiotics are processed in the body, their effect on an individual’s microbiological ecology, and studies of treatment efficacy and effectiveness.
- Enhanced surveillance on the incidence of infections, the way they are currently treated, clinical outcomes, and the influence of antimicrobial resistance, so we can better know where we are headed and model the effect of any possible changes in practice. Data will need to be clinically useful and better used in informing clinical decision-making, clinical guidelines and policy development.
- Associated costs and cost effectiveness.
- Improved ways of achieving translating new, robust evidence in clinical care in a wide range of settings internationally.
- Improving prevention of infections through changed lifestyle of individuals and communities, better farming methods, improved immunization and reduced opportunities for transmission.
- Enhanced access to effective antibiotics for those who will benefit and better ways of curtailing use where they are not effective
- How different classes of antibiotics, infection related strategies, and antibiotic use in humans and animals interact to produce both beneficial and unwanted outcomes. We need to see the world in an integrated, systemic way.

### Antibiotics: Proudly International, Multidisciplinary, Bold and Eclectic

To achieve these broad aims of biomedical innovation and antimicrobial stewardship, we will need the integrated perspectives of the clinical, mathematical, social, veterinary, economic, marketing, and policy sciences to first better understand the complexity of the culture and consequences of antibiotic use and human behavior in relation to infections, and then to develop, implement and evaluate the required innovations. 

So ***Antibiotics*** aims to bring together the relevant disciplinary perspective, including user perspectives, from around the world to contribute additional fresh impetus within the field. We will publish not only traditional research but also innovative, speculative perspectives, provided the criterion of scientific rigor is always met. We will welcome a broad range of research methods, so long as these are rigorous and appropriate to the research question. We will be nimble, fair and effective. We aim to become the leading and most vibrant open access journal in the field. 

### Antibiotics: A Journal with a Values Based Mission

Humanity faces a complex future. The challenges around preserving and developing antibiotic effectiveness are a microcosm of the challenges to humanity’s very existence; how do we maximize benefit from human ingenuity without destroying our future through failing to address the consequences of our current hubris? Good science and its timely, effective dissemination will contribute to this broad mission. With your support, this exciting new journal will rise to the challenge! *Antibiotics* therefore warmly welcomes researchers, users and readers, from every corner of the globe, who care about preserving and enhancing the miracle of antibiotics.

